# A Plea for a More Conservative Treatment of Appendicitis1Read at the 36th annual meeting of the Medical Association of Central New York, held at Auburn, September 22, 1903.

**Published:** 1904-01

**Authors:** F. W. Higgins

**Affiliations:** Cortland, N. Y.


### A Plea For a More Conservative Treatment of Appendicitis.[1]

By F. W. HIGGINS, M. D., Cortland, N. Y.

THE writer is well aware that he has chosen the unpopular side of the controversy in regard to appendicitis. Indeed, so many papers are being presented on the surgical treatment of this disease that one hesitates to bring forward the subject at all. My only excuse is that from cases recently seen I have concluded that it was necessary not to forget the grain of salt which must be taken with the extravagant statements of some of our operators.

Appendicitis is a surgical and a medical disease. In its consideration there is a radical and a conservative view. In this paper I have chosen to emphasise the conservative and medical treatment. I believe I yield to no one in my belief of the importance of surgical relief in proper cases. I do not hesitate to operate myself at once when the case demands. Still I believe it is timely to enter a plea for a more conservative treatment. In most of the discussions on this subject which are repeated at our medical meetings the burden is much the same. We are told that appendicitis is no longer a medical disease. As soon as a patient has a pain in his abdomen the case is to be considered as appendicitis and a surgeon to be called in. No time is to be lost. A leucocyte count is criminal, for the operation is delayed by so many minutes.

The entire fatality of the cases which die after the operation is to be laid at the door of the physician who did not call in the surgeon soon enough. Diagnosis is not a matter for consideration; the diagnosis is to be made while on the way to the patient with instrument case in hand, in fact the diagnosis is to be made when you hear the telephone bell ring. Or, to quote from their writings instead from memory,—"every case promising or unpromising, should be treated by surgical operation at the earliest possible moment." (Murphy. Sajous's Annual Analyt. Cyc., I., 479.) A reaction from this ultra and fantastic position is bound

---

1. Read at the 36th annual meeting of the Medical Association of Central New York, held at Auburn, September 22, 1903.

to come. Those who are now doing two or three operations daily will themselves become more conservative after a few years. But it behooves us who compose the rank and file of the profession,—the ordinary practitioners,—now to take sober second thought that we be not carried off our feet by those who are bulling the appendicitis market.

Pathologists are apt to become pessimistic. Who that has seen a series of appendices, secured postmortem or by operation, swollen, red, and sometimes adherent to surrounding organs, might not at first blush concur with the oft repeated statement of the surgeon to the family, that these were removed just in time to save life, and that the case would surely have proved fatal without an abdominal section. But other inflammations, equally severe, undergo perfect resolution. We do not cut away typhoid ulcerations which are just as marked as those of the appendix. A phlegmonous inflammation of the external auditory meatus looks as bad as the majority of removed appendices, but it gets well much better without an incision than with one. One does not operate now on all cases of lacerated cervix. The statistics of recovery without operation are very difficult to obtain. Most of us have too limited experiences in private practice to give a table that would not be worthless. Hospitals publish their operated cases but not so often the percentages of cure treated medically.

Treves (*Brit. Med. Joun.*, June 28, 1902,) in a recent carefully prepared and judicial paper, says that the mortality in medically treated cases is 5 per cent. If one takes account of slight and doubtful attacks this would be, I believe, about correct. If only well-developed cases be considered my own experience would be that it is about 10 per cent. General peritonitis is not the only fatal complication. Infected thromboses, metastatic abscess and cardiac infection, such as occur in other acute germ diseases, prove fatal in operated and nonoperated cases. Treves says that the mortality for operated cases in an acute attack is 20 per cent. Deaver, who is having as large an experience as anyone in this country, has had over 15 per cent.

Some of the would-be authorities on appendicitis give very misleading figures. Let me quote: "The surgical death-rate in acute and chronic appendiceal cases, without abscess, is a fraction of 1 per cent. at the hands of several American surgeons, and it is believed from classified data that the eventual death-rate in appendicitis cannot be much less than 25 per cent." (Morris, *Med. Times,* April, 1898). Uncritical reading of such a statement would leave the impression that 24 per cent. of cases of appendicitis could be saved by several American surgeons. Compare with this estimate the actual results reported by Richardson & Brewster. (*Boston Med. and Surg. Journ.*, July 14, 1898.) Of 464 acute cases, 284 were operated with 63 deaths, a mortality of 21 per cent. Out of 180 cases treated medically 31 died, a mortality of 17 per cent. They justly remark that some of the cases treated medically might have recovered by operation. On the other hand, some of the operations were probably unnecessary.

I am well aware that the figures mean little, but they are the best we have. Cases operated on in the interval have a slight mortality, perhaps less than 2 per cent. by skilled operators. If one takes out the healthy appendix, as some do in order to swell their statistics, the percentages may not be greater than 1 per cent. I am well aware of an argument in favor of radical operation in all cases, which is the liability of recurrence in cases which recover without operation. But the liability to hernia in the cicatrix, the secondary operations for adhesions and the recurrences which have been found even after operative interference go far to offset this argument. I have now under observation a letter carrier, who had a well-marked appendicitis in May, 1898. For six months afterward he was tender in the right iliac region. Since that time he has done his work with no recurrence or symptoms. About a year later I saw a rugged middle-aged carpenter in consultation. The symptoms of appendicitis were plain. We prepared for operation the next morning. At that time the patient was so much better that we waited further developments. He has been at work every day since. One patient of mine, a working man, had a sharp appendicitis in 1893. We deferred operation from day to day. He recovered without surgical intervention and is now perfectly well, never having had a recurrence.

The question whether recurrences have happened is difficult to settle in many cases. We most often decide from a description by the patient of symptoms had years before. To present some slight basis of facts myself, however, I have obtained the experience of five of the physicians in our place who have been in active practice for several years. Combined with my own experience 21 per cent. of our cases have recurred. I believe appendicitis to be an infective inflammation often caused by traumatism or extension from the cecum. Resolution may be as complete as in any other structure with no predisposition to future attacks. But if a second attack occurs showing that there is probably an anatomical change produced, an interval operation is indicated. It is a close parallel to tonsillitis. Here we must evacuate pus if a retrotonsillar abscess forms, but a radical tonsillotomy is to be avoided in the presence of active inflammation. This would incur too great risk of opening up fresh avenues to bacteria and their toxins, which are now being walled off.

Permit me in few words to sketch what I believe should be the conservative treatment of this disease. Rest is all important. No cathartics should be administered, but large colonic flushings with salt and water or linseed oil should be given at the very beginning of the attack. Hardened feces retained in the head of the colon or a nidus of bacterial culture may be thus washed away. Food should be interdicted absolutely in order that peristalsis shall be reduced to a minimum. Frequent and deep pressure over McBurney's point must be avoided. I am quite sure I knew a resolving appendicitis lighted up and the young lady's life sacrificed by a consultant who felt he must palpate the diseased appendix. A careful watch of the case must be instituted with the help of a trained nurse. If an abscess forms it must, of course, be opened as should a collection of pus elsewhere. In most cases it is better to leave the appendix, which forms a part of the abscess wall, to take care of itself under drainage.

The signs of abscess formation are not absolute but must be considered carefully as would any other internal disease. As helpful, may be mentioned slight dulness and leucocytosis. Conclusions drawn from attempts to palpate the appendix are entirely fallacious. If pus does not form too rapidly resolution is to be expected. I know of no disease, however, which is to be watched with greater anxiety and care. We are told that this uncertainty could have been avoided by an operation during the first 24 hours. But at least 60 per cent. of the appendices so removed would be normal. The only symptoms present at that time cannot be distinguished from a colic due to indigestion.

The strongest argument set forth against delayed operation has not yet been mentioned. It is that the appendix may rupture early into the free abdominal cavity with the formation of no limiting inflammation. This does undoubtedly happen, but is of very rare occurrence. It would most often be announced by a general peritonitis before any symptoms would have called the attention of surgeon or physician to the presence of the larva disease. In the face of a developed general peritonitis from such an accident, abdominal section is of little use. By far the best results are obtained from following Ochsner's plan of treatment, namely, avoiding all cathartic or other medication, washing out the stomach, and abstaining from all food by the mouth. These measures have proven of the utmost benefit in two recent cases where I have recommended the plan. Should the general peritonitis subside as it sometimes does under this treatment, any local collection of pus or a diseased appendix may be later removed.

To summarise: my opinion is that our cases of appendicitis should be treated as medical cases, carefully watched, operated when indications demand, after careful unprejudiced consultation. Interval operations should be recommended after a second attack. In this way I believe the largest percentage of our cases will be preserved in life and returned to usefulness.

DISCUSSION.

Dr. N. S. Jacobson, Syracuse: It does not seem to me a paper of this kind should go unchallenged in a body like our own. I think that anything but a conservative statement of the situation was the introduction of the paper, namely—that it is customary that the diagnosis should be made on the way to the patient, or the decision to operate made before the patient is seen. I am sure that all of us believe in conservatism, but our definitions of conservatism may differ. My own idea of conservatism is that which saves life, and I believe that which saves life is the most conservative measure to adopt in any case. I am sure those of us who are brought face to face with cases of appendicitis that are surgical cases (cases that have been treated as medical cases, and turned over to us when all medical hope has been abandoned, and sometimes when all other hope has to be abandoned also), have every reason to believe that the procedure which has antedated our connection has been anything but a conservative one.

I do not think any one here will claim that the mild cases of appendicitis, which show no acute disturbances during the first 24 hours, which show a disposition to subside after 24 hours, that the second day show improvement, and the third still more, demand any operation during that first attack. The very statement of the reader of the paper, that after the second occurrence of such a manifestation he would advise an operation, shows for itself that he cannot take the position that appendicitis is a medical disease. Now, I feel sure with that statement we can agree, namely,—that in those mild cases which have twice recurred, there is evidence of a lasting lesion which demands attention, and therefore that class should be treated surgically; but you and I, Mr. Chairman, have had the opportunity to see those cases which did not allow 24 hours delay. We have seen cases where four or five hours after the first manifestation, with no attack before, there has been a general widespread infection, and the patient has been practically moribund, or beyond help when the surgeon is called in.

I know of no condition which demands more careful investigation than that disturbance of the appendix which we speak of as appendicitis, and I believe a careful examination of a case that shows progression after the first 24 hours, indicates that it is not a medical but a surgical disease. I believe that those cases of appendicitis which show subsidence of all symptoms after 24 hours should not be operated upon, and the very fact that every surgeon has occasion to operate during the quiescent period shows, and the fact that he recommends it also shows, that he does not himself believe that all the cases of appendicitis demand an operation during the attack; but, I think, the author of the paper was most unfortunate in comparing an infected tonsil with an infected appendix, and in making the statement that it was as unwise to cut through an infected appendix as it was an infected tonsil. The conditions are absolutely different. When you remove a diseased appendix you go outside of it, but how often can you go outside of the tonsil? You would simply be cutting through tissue, while many times we are able to remove an infected appendix and still carry our work outside and into tissue not so badly involved. But it seems to me that the more we have occasion to see this disease, the more we are satisfied that the large percentage of cases demand surgical operation; and I think the poorest argument that can be presented is the word of statistics. I do not see how you are going to compare these cases on the basis of numbers, or so-called statistics that are gathered by surgeons in hospitals.

Those of us who have surgical work to do in hospitals know it is the dumping ground of all kinds of cases, and we have to receive for operation cases advanced, cases acute, cases curious, and all kinds of cases that come into the surgical ward for operation. Would you think of comparing the results of such cases with the result of a class of cases that have been treated medically? Because they have not been acutely infected, and because they are not gangrenous, and because they do not involve the general peritoneum, shall we say they are to be compared, when the only estimate is one of figures, one of so-called results?

It is utterly impossible to compare results in such a case, but I do know that, given a case of appendicitis, if it is strictly a case of appendicitis, no matter how infected it is, provided there is no development outside of the appendix, the chances are that every case operated on will improve greatly. The death-rate is not due to the appendicitis, but it is due to the fact that the disturbance has spread out of the appendicitis and has involved the peritoneal cavity. So, I believe that it is fair to say that the conservative procedure is, that in progressive appendicitis, progressing into the second and third day, without signs of abatement, surgical and not medical, and if we are to treat a class of cases as purely medical, it shall be that class which shows marked improvement on the second day.

